# Treatment strategies for recurrent dislocation following total hip arthroplasty: relationship between cause of dislocation and type of revision surgery

**DOI:** 10.1186/s12891-023-06355-4

**Published:** 2023-03-29

**Authors:** Takashi Toyoda, Kenichi Oe, Hirokazu Iida, Tomohisa Nakamura, Naofumi Okamoto, Takanori Saito

**Affiliations:** Department of Orthopaedic Surgery, Kansai Medical University, 2-5-1 Shinmachi, Hirakata, 573-1010 Osaka Japan

**Keywords:** Cement, Constrained cup, Dislocation, Revision, Total hip arthroplasty

## Abstract

**Background:**

There are many therapeutic options for dislocation following total hip arthroplasty (THA). The aim of this study was to evaluate the results of revision surgery for dislocated hips.

**Methods:**

Between November 2001 and December 2020, 71 consecutive revision hip surgeries were performed at our institution for recurrent dislocation following THA. We conducted a retrospective study of all 65 patients (71 hips), who were followed for a mean of 4.7 ± 3.2 years (range, 1–14). The cohort included 48 women and 17 men, with a mean age of 71 ± 12.3 years (range, 34–92). The mean number of previous surgeries was 1.6 ± 1.1 (range, 1–5). From intraoperative findings, we created six categories of revision hip surgery for recurrent dislocation following THA: open reduction and internal fixation (2 hips); head change or liner change only (6 hips); cup change with increased head size only (14 hips); stem change only (7 hips); cup and stem change (24 hips); and conversion to constrained cup (18 hips). Prosthesis survival was analyzed by the Kaplan-Meier method, with repeat revision surgery for re-dislocation or implant failure as the endpoint. A cox proportional hazards model was used for risk factors of re-revision surgery.

**Results:**

Re-dislocation occurred in 5 hips (7.0%) and implant failure in 1 hip (1.4%). The 10-year survival rate was 81.1% (95% confidence interval, 65.5–96.8). A Dorr classification of “positional” was a risk factor for re-revision surgery due to re-dislocation.

**Conclusion:**

Clear understanding of the cause of dislocation is essential for optimizing revision procedures and improving the rate of successful outcomes.

## Background

Although total hip arthroplasty (THA) has made remarkable advances since its first introduction [1], even the most up-to-date surgical techniques are still associated with complications, and with the steady rise in the number of THAs being performed, revision surgeries to manage those complications are expected to increase [2, 3]. In that regard, dislocation is currently the primary reason for revision surgery in the US [4, 5]. A study of 417,687 THAs from the Nordic Arthroplasty Register Association database showed a 0.5% incidence of hip dislocation after primary THA [6], while a systematic review of 4,656 revised THAs showed a 9.0% incidence of dislocation following revision [7], and the incidence of re-dislocation following revised THA for dislocation was 18–39% [8, 9]. This situation is extremely challenging for surgeons.

The specific technique for revision surgery should be selected depending on the cause of dislocation in that particular patient. Typically, revision surgery utilizes one of several types of constrained cup. However, this choice requires careful consideration of postoperative joint function and implant survival rate. The dual-mobility cup, although offering an extraordinary advance that can provide patients with greater jumping distance and better range of motion following THA [10, 11], cannot prevent all dislocations. Rather than focusing on the newest and most advanced implants, the most important points in revision surgery for dislocation are to conserve soft tissue and to optimize implant repositioning. Such repositioning includes not only correction of alignment or impingement, but also reconstruction at the level of the true hip center, for example, lowering a high hip center if needed. These goals can be accomplished in many cases by adjusting the stem version or the depth of insertion. If the soft tissue has undergone extensive damage and is incapable of fully stabilizing the prosthetic joint, a constrained cup should be used. Unfortunately, only a few studies have focused on surgical techniques appropriate for specific causes of dislocation [9, 12–14]. The present study was thus designed to evaluate the results of revision surgery for dislocated hips and to identify risk factors for re-revision after revised THA due to recurrent dislocation.

## Methods

### Study design and patients

Between November 2001 and December 2020, 71 consecutive revision hip surgeries for recurrent dislocation following THA were performed by four experienced surgeons at our institution. We conducted a retrospective study of all 65 patients (71 hips), who were followed for a mean of 4.7 ± 3.2 years (range, 1–14 years). The subject population consisted of 48 female patients (52 hips) and 17 male patients (19 hips), with a mean age of 71 ± 12.3 years (range, 34–92 years) at the time of surgery. Dislocation had followed primary THA in 45 hips (63%) and revised THA in 26 hips (37%). The mean number of previous surgeries was 1.6 ± 1.1 (range, 1–5).

Before surgery, anteroposterior and lateral radiography and computed tomography (CT) were performed to check for implant malposition, raised bone, fractures, and non-union. Joints were examined under traction or motion using X-ray imaging without anesthesia to assess soft tissue imbalance and impingement and to identify the cause of dislocation. Gluteus medius failure was diagnosed if the hip dislocated readily in response not only to ordinary traction, but also to the application of lateral stress with the hip in adduction and lateral or backward stress with the hip flexed at 90 degrees. Causes of revision hip surgeries for recurrent dislocation following THA were categorized based on Dorr classification [15]: type I (positional) in 2 hips, type II (soft tissue imbalance) in 58 hips, and type III (component malposition only) in 11 hips (Table 1). Dislocations were classified as type III if they were caused by cup migration and loosening over time or by the implant itself (thick stem neck, etc.).

**Table 1:** Preoperative patient characteristics

Characteristics	Value
**Number of hips**	71
**Age at surgery (years), mean ± SD (range)**	71 ± 12.3 (34 − 92)
**Sex, male:female**	19:52
**Follow-up period (years), mean ± SD (range)**	4.7 ± 3.2 (1 − 14)
**Duration between dislocation and revision surgery**	
<2 year ≥2 year	40 31
**Direction of dislocation**	
Anterior Posterior Unknown	32 16 23
**Previous surgery (times)**	
1 (Primary THA) 2 ≥3	45 17 9
**Previous approach**	
Anterolateral Posterior Greater trochanteric osteotomy Unknown	41 17 1 12
**Previous operating institution**	
Our hospital Other hospital	22 49
**Previous fixation method**	
Cement Cementless Hybrid	55 12 4
**Causes of revision surgery for recurrent dislocation** ^**a**^	
Type I (positional) Type II (soft tissue imbalance) Type III (component malposition only)	2 58 11

THA, total hip arthroplasty; SD, standard deviation

^a^ Causes for recurrent dislocation were categorized according to Dorr classification

Our institutional review board (2,021,153) approved this retrospective cohort study. Each patient provided informed consent for data included in the published findings.

## Revision hip surgery

**Fig. 1:**
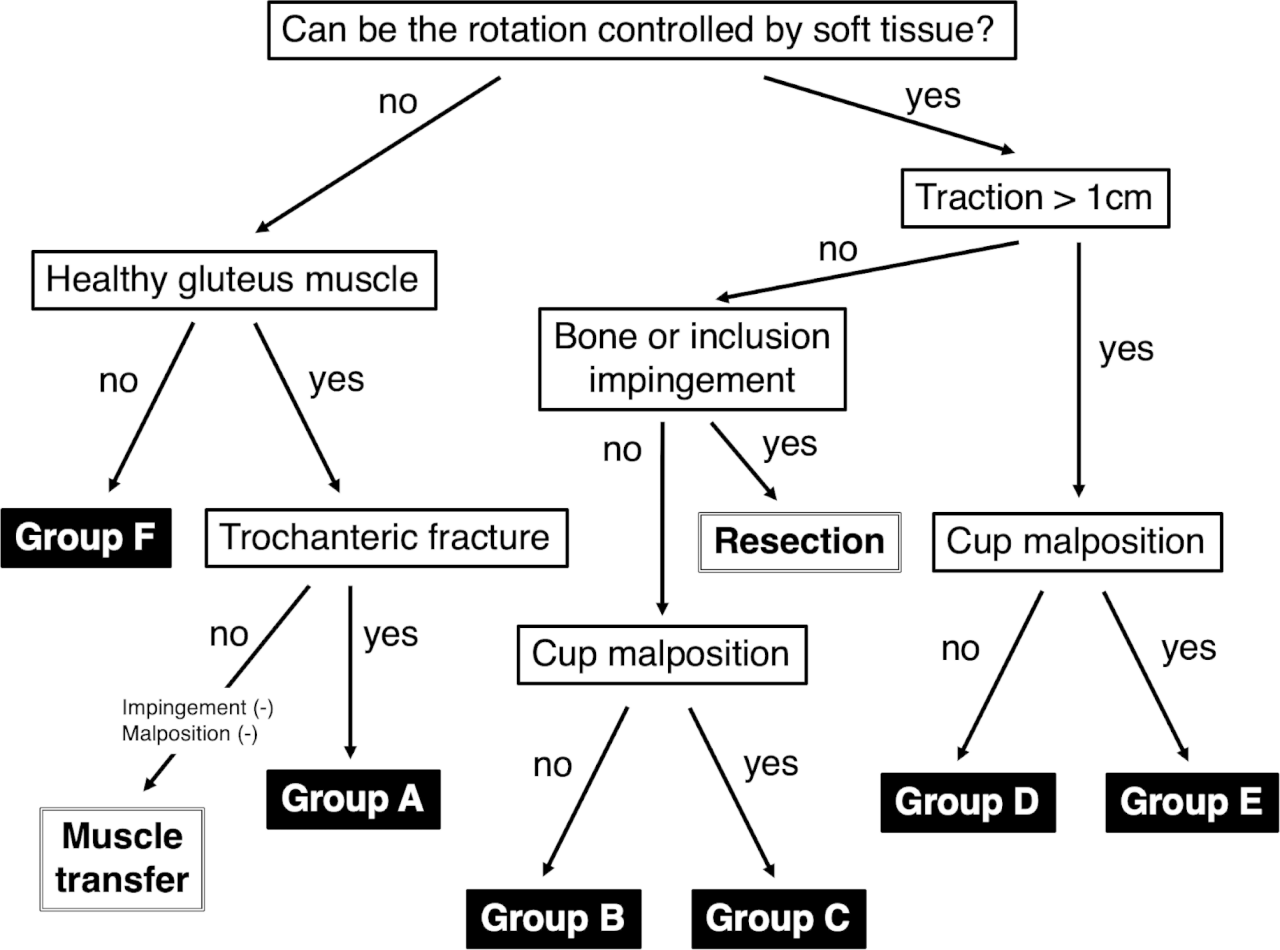
Algorithm for management of recurrent dislocation following total hip arthroplasty. Group A, open reduction and internal fixation; Group B, head change or liner change only; Group C, cup change with increased head size only; Group D, stem change only; Group E, cup and stem change; and Group F, conversion to constrained cup

**Fig. 2:**
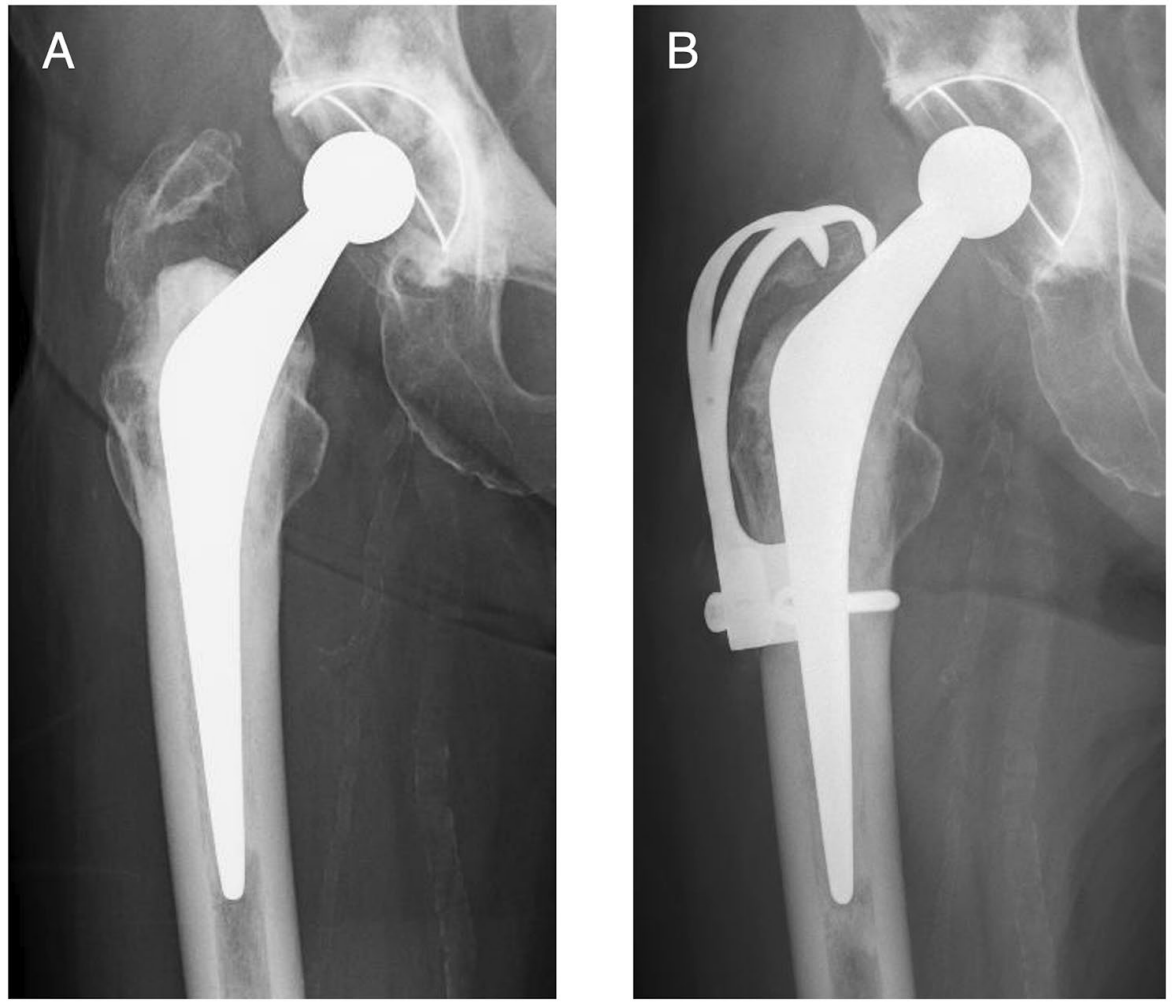
Anteroposterior radiographs of a 70-year-old woman who underwent primary total hip arthroplasty 2 years ago. **A**: Post-operative trochanteric fracture developed, and 3 months after surgery the patient experienced recurrent dislocation of the hip due to gluteus medius failure (type II). **B**: Open reduction and internal fixation were performed. It has now been 1.5 years since the revision surgery (Group A)

**Fig. 3:**
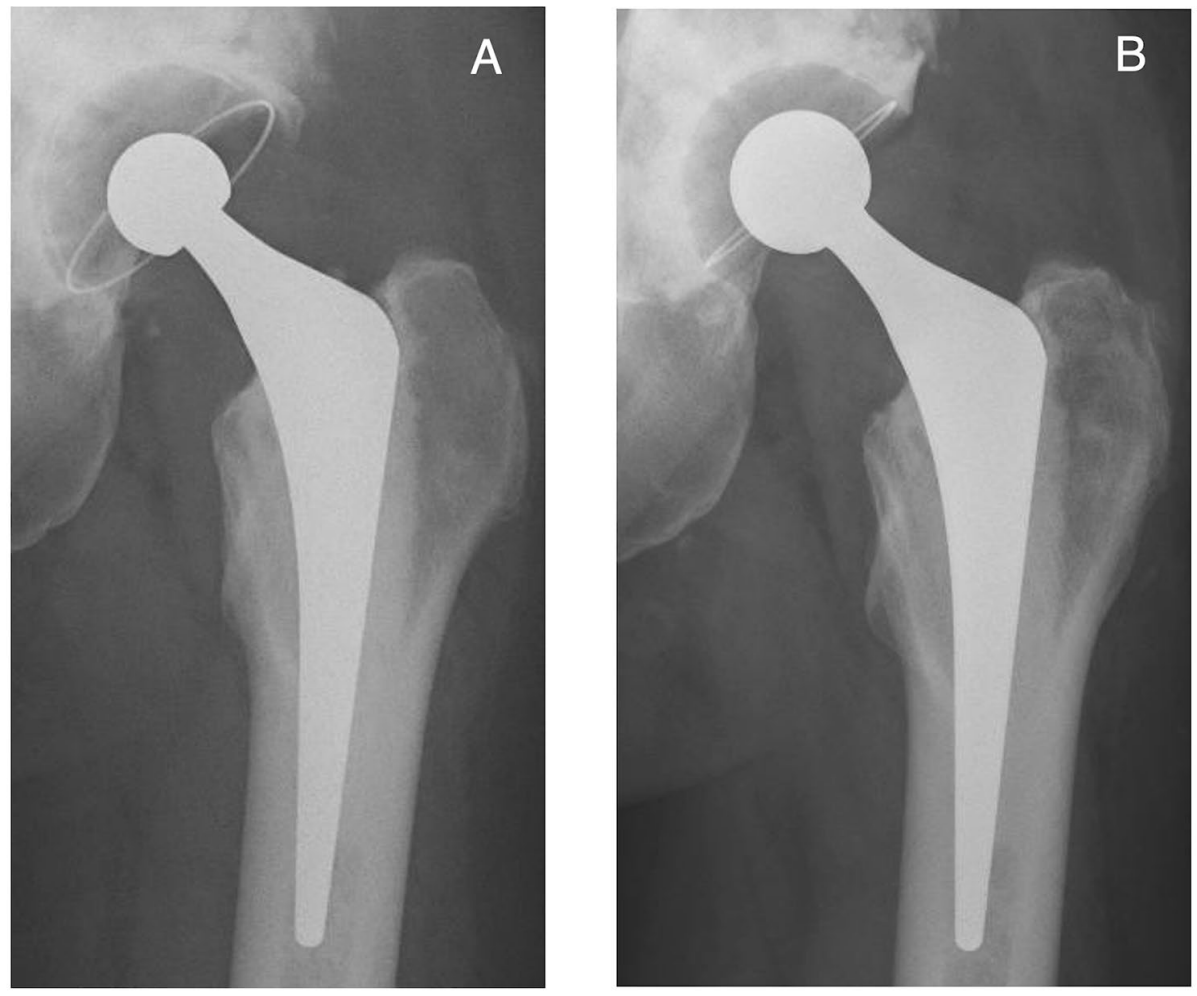
Anteroposterior radiographs of a 49-year-old woman who underwent primary total hip arthroplasty (THA) 2.5 years ago. **A**: Two years after surgery, the patient experienced recurrent dislocation because of cup retroversion (type III). **B**: Cup alignment was corrected, and the femur head was changed from 22.225 to 26 mm. It has now been 4 years since the revised THA (Group C)

**Fig. 4:**
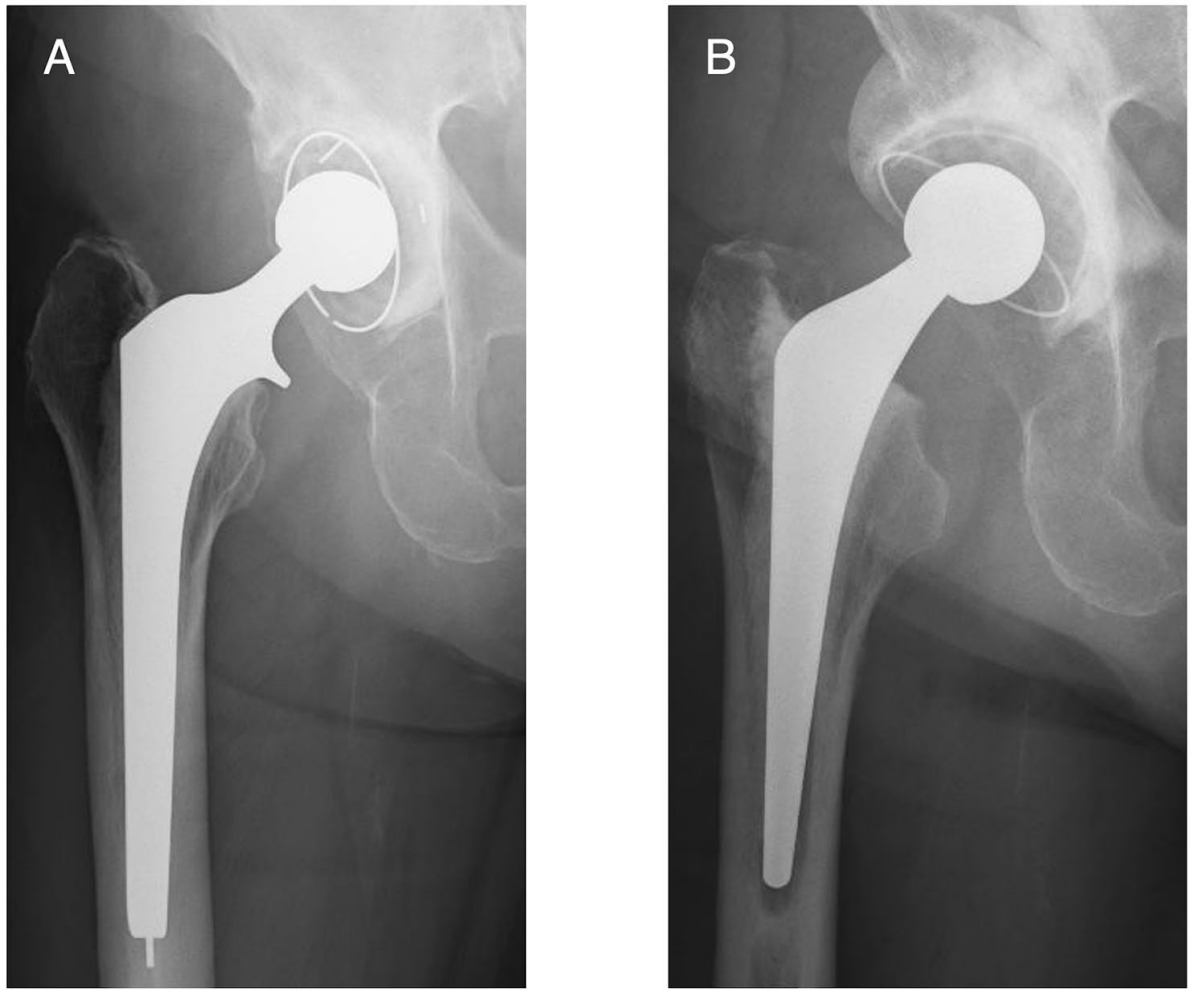
Anteroposterior radiographs of an 82-year-old woman who underwent primary total hip arthroplasty (THA) 7 years ago. **A**: She experienced recurrent dislocation caused by cup malposition (type III). **B**: Cup alignment was corrected, and the stem was replaced using the cement-in-cement technique. It has now been 2 years since the revised THA (Group E)

**Fig. 5:**
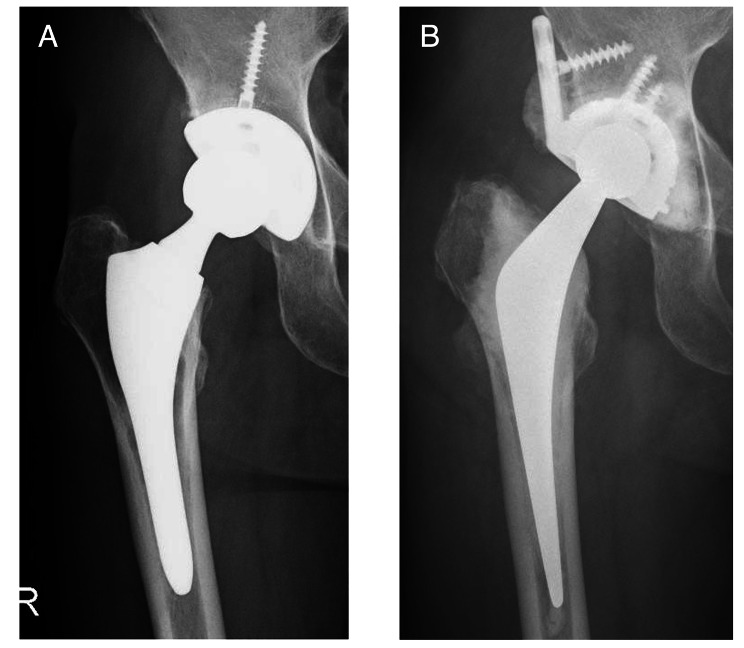
Anteroposterior radiographs of a 79-year-old man who underwent primary total hip arthroplasty (THA) 2 years ago. **A**: Three months after surgery, the patient experienced recurrent dislocation caused by soft tissue imbalance resulting from the posterior approach (type II). **B**: Because of insufficient tension in the soft tissue, a constrained cup was used for reversion. It has now been 4 years since the revised THA (Group F)

Based on intraoperative findings from revision hip surgery that used implants for recurrent dislocation following THA, we grouped our target hips into 6 categories of revision hip surgery for recurrent dislocation following THA: group A, open reduction and internal fixation (ORIF); group B, head change or liner change only; group C, cup change with increased head size only; group D, stem change only; group E, cup and stem change; and group F, conversion to constrained cup (Fig. 1). In Group A, ORIF was performed for dislocation caused by greater trochanteric fracture associated with gluteus medius deficiency (Fig. 2). In group B, the head was changed to a longer neck length, or the liner was changed. In Group C, the head was changed to a larger diameter, and the cup was changed to match the new head (Fig. 3). In Group D, stem version or depth of insertion was adjusted by changing stems using the “cement-in-cement” technique [16]. In Group E, both cup and stem were changed, using the same techniques as for Groups B, C, and D, using a conventional cup (Fig. 4). In Group F, the cup was converted to a constrained cup (Fig. 5). This technique was used in patients who had either insufficient soft tissue tension or impingement that was unlikely to be managed by the techniques used in Groups A–E. In some cases, stem version or depth of insertion were adjusted by changing stems, as was done in Group D. In some patients in Groups D–F, the stem was replaced to make the legs of equal length.

The transgluteal approach in the lateral position was used in all patients. The implant was deployed, and a direct visual check was made to confirm that there was no component failure, component malposition, or impingement, and that no problems with soft tissue were anticipated. After this visual check, the details of treatment strategy were determined. In revised THA, a cemented cup and stem were implanted. In the revision surgery for hips with greater trochanteric fracture, osteosynthesis was performed by implanting a trochanteric claw plate (CMK Trochanteric plate, Zimmer Biomet Holdings Inc., Warsaw, IN). This revision procedure was also used in Group A hips. Our institution does not routinely perform ORIF as first-line treatment, even in patients with greater trochanteric fracture, because in many cases the fracture does not lead to recurrent dislocation, and in our experience the trochanteric claw plate used in ORIF can itself be a cause of long-term pain in some patients. Revision of the cup was performed using a K-MAX CLHO flanged cup with 26-mm head (Kyocera Medical, Osaka, Japan). If necessary, structural allografts and KT plates (Kyocera Medical) were used for massive bone defects [17, 18]. For conversion to a constrained cup, we used the Physio-Hip System Reconstruction Cup (Kyocera Medical), and for stem revision surgery, we used an SC stem (Kyocera Medical) in 14 hips, an HS-3 stem (Kyocera Medical) in 9 hips, a C stem (DePuy International, Leeds, United Kingdom) in 8 hips, a PHS long stem (Kyocera Medical) in 2 hips, and an Exeter stem (Stryker Orthopaedics, Mahwah, NJ) in 2 hips. Long stems were used if a normal stem was considered potentially unstable because of bone fragility or defect. A dislocation test was performed using a trial stem and head to determine the stem version and the depth of insertion. All components were fixed with Endurance Bone Cement (DePuy CMW, Blackpool, United Kingdom) using a third-generation cement technique. Full weight-bearing was permitted as soon as possible, although the use of a cane was encouraged for up to 3 months.

## Follow-up protocol

Postoperative follow-ups were performed at 2 weeks, 3 months, 6 months, 1 year, and annually thereafter. A retrospective analysis was conducted by 2 blinded orthopedic surgeons. The causes of recurrent dislocation were noted, optimal revision strategies for each were assessed. Postoperative complications, including re-dislocation, implant breakage, implant loosening, periprosthetic fracture, and periprosthetic infection were recorded.

## Statistical analyses

For prosthesis survival, we used the Kaplan-Meier method with 95% confidence intervals (CIs). The study end points were repeat revision surgery for re-dislocation, implant breakage, or implant loosening. Univariate logistic regression analysis using a Cox proportional hazards model was performed for risk factors of re-revision surgery following re-dislocation, implant breakage, or implant loosening. Patient variables included age, sex, number of previous surgeries, implant (cement or cementless), Dorr classification, and revision hip surgery. Data were analyzed using SAS 9.2 (SAS institute Inc., Cary, NC).

## Results

Table 2 shows the relationships between cause of recurrent dislocation and revision hip surgery for each cause. The mean number of previous surgeries was 2.0 ± 1.2 (range, 1–5) for patients who underwent constrained cup implantation and 1.4 ± 0.7 (range, 1–4) for patients who did not receive a constrained cup. Of those converted to a constrained cup, the stem was also changed in 4 hips and the existing stem was retained in 14 hips. Among 35 total cases of stem change (all 7 hips in Group D, all 24 hips in Group E, and 4 hips in Group F), the original implant had a cemented stem in 32 hips (91.4%). Cup malposition requiring revision because of recurrent dislocation was noted in 38 hips (Groups C and E), including cup retroversion in 9 hips and cup vertical placement in 29.

**Table 2:** Relationship between dislocation causes and revision hip surgeries for recurrent dislocation

Revision hip surgery	Type I (n = 2)	Type II (n = 58)	Type III (n = 11)
**Group A: Open reduction and internal fixation**	-	2	-
**Group B: Head change or liner change only**	-	2 + (1)	3
**Group C: Cup change with increased head size only**	(1)	10	3
**Group D: Stem change only**	-	6	1
**Group E: Cup and stem change**	(1)	18 + (1)	3 + (1)
**Group F: Conversion to constrained cup**	-	18	-

Figure in parentheses indicates the number of re-revision surgeries for re-dislocation

Causes for recurrent dislocation were categorized according to Dorr classification

Repeat revision surgery was performed in 9 patients for the following indications: re-dislocation in 5 patients (7.0%); periprosthetic infection in 3 patients (4.2%); and implant breakage in 1 patient (1.4%). Details are shown in Table 3 for 5 patients who underwent re-revision surgeries for re-dislocation. All patients had undergone 1 previous revision surgery (revision after primary THA). Of the 5 patients who had re-revision surgery, 4 suffered from mental health issues. Dislocation recurred following the re-revision surgery in 2 of those patients, but no further surgical revisions were performed. Dislocation rates following re-revision surgery for recurrent dislocation (Dorr classification) were 100% (2/2 cases) for type I, 3% (2/58) for type II, and 9% (1/11) for type III. One patient required re-revision surgery for implant breakage, followed by additional surgery 2.4 years after the re-revision. That patient had previously undergone 5 surgeries.

**Table 3:** Patients undergoing re-revision surgery for re-dislocation in this study

Case	Age/Sex	Dorr classification	Previous THA (times)	Revision surgery	Duration from previous THA to revision surgery (years)	Re-revision surgery	Comment
1	52/M	Type I	1	Group C	0.7	Conversion to hemiarthroplasty	Drug addiction
2	48/M	Type I	1	Group E	4.4	Only stem change	Schizophrenia
3	72/F	Type II	1	Group E	0.5	Conversion to constrained cup	Lack of understanding
4	75/F	Type II	1	Group B	0.4	Conversion to constrained cup	(-)
5	83/F	Type III	1	Group E	4.5	Conversion to constrained cup	Dementia

THA, total hip arthroplasty

With repeat revision surgery for re-dislocation or implant failure as the endpoint, the 10-year survival rate was 81.1% (95% CI, 65.5–96.8); that rate was 72.8% (95% CI, 58.9–94.2) after primary THA and 90.3% (95% CI, 82.0–99.5) after revised THA. Constrained cups were used in 26.7% (12/45 cases) for primary THA and 23.1% (6/26 cases) for revised THA. Univariate logistic regression analysis for risk factors of re-revision surgery due to re-dislocation and implant breakage is shown in Table 4. Patient position was identified as a risk factor (Dorr type I, hazard ratio = 17.83 vs. type II, *p* = 0.0025).

**Table 4:** Risk factors in univariant logistic regression analyses for re-revision surgery due to re-dislocation and implant breakage

	No reoperation (n = 65)	Reoperation (n = 6)	Hazard ratio	95% CI	*p* value^a^
**Average age**	71.2	66.7	0.99	0.93–1.05	0.7073
**Male (%)**	17 (26.1%)	2 (33.3%)	1.01	0.18–5.54	0.9943
**Duration to revision**					
<2 year ≥2 year	37 28	3 3	control 1.25	(-) 0.11–14.72	(-) 0.8562
**Previous operation**					
1 2 ≥ 3	40 16 9	5 1 0	NA	NA	NA
**Fixation method**					
Cement Cementless Hybrid	49 12 4	6 0 0	NA	NA	NA
**Dorr classification**					
Type I Type II Type III	0 55 10	2 3 1	17.83 control 1.37	2.76–115.3 (-) 0.13–13.96	0.0025 (-) 0.7895
**Revision surgery**					
Group A Group B Group C Group D Group E Group F	2 5 13 7 21 17	0 1 1 0 3 1	0 1.71 0.49 0 control 0.56	0 0.17–17.62 0.05–4.77 0 (-) 0.06–5.37	0.9969 0.6537 0.5375 0.9963 (-) 0.6137

95% CI, 95% confidence interval; NA, not analyzed

^a^Cox proportional hazards model

## Discussion

The number of revision surgeries for primary THA is increasing, with dislocation being the No. 1 cause in the United Stated and No. 4 in the United Kingdom [2–5, 19]. Recurrent dislocation requires revision surgery, and when that surgery is revised THA, the subsequent incidence of re-dislocation can be as high as 18–39% [8, 9]. Dislocation also occurs in as many as 9% of patients after revised THA for other reasons [7].

In 1983, Dorr et al. originally classified causes of dislocation into the three categories of “positional”, “soft tissue imbalance”, and “component malposition” [15], and in 1998 they added a fourth category of “component malposition and soft tissue imbalance” [12]. Of the 55 dislocated hips that they reviewed, 44% (24/55) underwent dislocation-induced revision surgery, and the cause of dislocation was classified as “positional” in 15%, “soft tissue imbalance” in 19%, “component malposition” in 40%, and “component malposition and soft tissue imbalance” in 25%. Among our patients, we have found that that instances of “component malposition and soft tissue imbalance” are actually attributable to “soft tissue balance” alone, so for the current study, we subsumed the fourth category of “component malposition and soft tissue imbalance” within the category of “soft tissue imbalance,” and we used the original three Dorr categories to evaluate the results of our revision surgery. Our rate of re-revision surgery for “positional” dislocation was 100% (2/2 hips). Both of these cases suffered from mental health issues (one with schizophrenia and the other with drug addiction), and in retrospect, we should have focused more on each patient’s ability to comply with postoperative requirements when choosing our treatment strategy.

A Japanese multicenter study of 88 hips showed recurrent dislocation in 18% (16/88) [9], with abductor deficiency, which generally involves the use of a constrained cup in revision surgery, implicated in 44% (7/16) of those re-dislocations. In an unrelated study of 75 hips, Wera et al. also reported a 15% rate of re-revision surgery for re-dislocation (11/75 hips) [14]. That study used 6 categories for cause of dislocation: abductor deficiency (36%), cup malposition (33%), impingement (9%), stem malposition (8%), late wear (7%), and unsolved etiology (7%). Clearly, the results of revision surgery were poorest in patients whose dislocation was attributed to abductor deficiency. Interestingly, although the 11 failed procedures included 9 that had used a constrained cup, the rate of dislocation was higher in those patients whose revision was for a failed constrained cup. We limited our use of the constrained cup to only 18 of the 71 hips in our study, and none of those 18 experienced re-dislocations. This difference, which encourages careful consideration of when to select the constrained cup, could be usefully explored in further studies. A single-center study in the USA reviewed 156 hips and reported recurrent dislocation in 21% (33/156) [13]. The authors classified revisions into 4 categories by surgical procedure: isolated linear exchange (56%), acetabular component revision (33%), stem revision with liner exchange (5%), and both component revision (8%). The use of a constrained cup was associated with dislocation in 20.3% of hips and cup loosening in 8.5%. Although their report did not mention implant type, we assume that cementless THA was used in most cases, which could explain the higher percentage of hips undergoing isolated liner exchange and the lower percentage that had stem revision. Of our 35 hips that underwent stem change, 32 had cemented stems, which may explain why our results show stem revision to be relatively straightforward. Although a detailed discussion of the relative benefits of cemented and cementless THA is beyond the scope of this paper, these two previous reports clearly show an association between abductor deficiency (soft tissue imbalance) and high re-dislocation rate. Those circumstances appeared to require the use of a constrained cup in revision, but the resulting performance of the constrained cup was not always as favorable as desired.

In the present study, no patients required re-revision surgery due to re-dislocation using a constrained cup, although one repeated revision was needed for a broken implant. We chose a constrained cup for three of the five hips that underwent re-revision surgery due to re-dislocation. After considering the age of the patients, we chose not to use a constrained cup in the remaining two hips. Our overall survival rate for primary THA was slightly lower than for revised THA, despite near-equivalent usage of constrained cups. This similarity may occur because revision surgery is considered more technically demanding and is thus frequently assigned to more experienced surgeons. Although revision surgery should be optimized to best manage the cause of dislocation (Table 2), it is rarely simple – but always essential – to assess that cause before determining the most appropriate revision procedure. The current algorithm can be helpful (Fig. 1).

Surgeons should keep in mind that dislocation following THA can often be attributed to technical errors before or during surgery, including the selection of the approach or the type of implant. For example, large head implants have been widely used for primary THA in recent years, partly because they offer increased jumping distance and a larger oscillation angle. However, large-head implants (≥ 32 mm) are associated with high revision rates according to the Australian Orthopaedic Association National Joint Replacement Registry [20]. Charnley himself is known to have achieved a highly favorable rate of dislocation (0.5%) by using a femoral head 22.225 mm in diameter for THA [21], underscoring the importance of selecting surgical techniques that are gentle on soft tissue and of using femoral heads that are not excessively large. We use cement fixation for primary THA in all patients at our institution, and we generally choose a 22.225-mm diameter femoral head. In 18% of cases (Group C), we successfully addressed recurrent dislocation by changing the head to 26 mm in diameter. The cement-in-cement technique made it easy to replace stems, an important advantage of cement fixation (Groups D and E). By using this technique, which encourages supportive tension in the soft tissue, we were able to avoid the use of a constrained cup. Other researchers have also reported lower rates of dislocation for cemented THA [22, 23]. These findings suggest the selection of THA procedures that are gentle on soft tissue and that simplify future revision if required.

This study has some limitations. First, patients were retrospectively evaluated without a control group, and the follow-up period was limited to one year; continued follow-up will be required to establish long-term outcomes. The sample size was relatively small, involving only 71 individuals. Furthermore, surgeons in our hospital and associated hospitals usually select the anterolateral approach, so that approach was widely used in the current study, which may have affected patient outcomes. The relationship between surgical approach and dislocation should be explored in further studies. Second, we evaluated patients who underwent re-revision surgery for recurrent dislocation, but that evaluation excluded cases of dislocation that did not result in revision surgery. Third, we did not objectively evaluate soft tissue; instead, we used a constrained cup if preoperative X-ray images showed potential rupture in the gluteus medius or if intraoperative findings suggested that soft tissue would be unstable with a conventional cup. In addition, revision because of cup malposition involves complex decision-making. Cup malposition cannot be defined by X-ray imaging and CT alone, but must be confirmed intraoperatively to determine if revision is called for, and even if the cup is malpositioned, the femur may not dislocate if the stem is well-positioned, in which case revision is not required. In Group E, the most common cause of dislocation was soft tissue imbalance, although cup malposition was noted in 24 hips.

## Conclusion

We reviewed the results of revision hip surgeries based on the cause of dislocation in 71 patients and found that re-revision surgery due to re-dislocation occurred in 7.0% (5/71). The primary risk factors for re-revision surgery were the patient taking a position that exceeded the mechanical stability of the implant (type I in the Dorr classification of causes) and mental health issues. If the implant was cemented in the primary THA, proactive re-conversion of the stem was a feasible option. We attribute favorable results in Groups D and E to that factor. We were also able to resolve re-dislocation in some patients (Group C) by exchanging the 22.225-mm diameter femur head for a 26-mm head. Our findings suggest that more options are available for revised THA when the implant was cemented in the original THA. We used a constrained cup in 25.4% (18/71) of cases. However, this type of cup is not always necessary when treating recurrent dislocation, and its use should be considered carefully with full attention to the patient’s condition and age. These findings emphasize the importance of assessing the cause of the dislocation when determining the most appropriate revision procedure.

## Declarations

### Available of data and materials

The datasets used and/or analyzed during the current study are available from the corresponding author on reasonable request.

## Abbreviations

THA: Total hip arthroplasty
ORIF: Open reduction and internal fixation
95% CI: 95% confidence interval.
